# 1-Diphenyl­methyl­ene-2-(9*H*-fluoren-9-yl­idene)hydrazine

**DOI:** 10.1107/S160053681000070X

**Published:** 2010-01-13

**Authors:** R. Archana, R. Anbazhagan, K. R. Sankaran, A. Thiruvalluvar, R. J. Butcher

**Affiliations:** a PG Research Department of Physics, Rajah Serfoji Government College (Autonomous), Thanjavur 613 005, Tamil Nadu, India; b Department of Chemistry, Annamalai University, Annamalai Nagar 608 002, Tamil Nadu, India; c Department of Chemistry, Howard University, 525 College Street NW, Washington, DC 20059, USA

## Abstract

In the title mol­ecule, C_26_H_18_N_2_, the 9*H*-fluorene unit is almost planar, as the cyclo­penta­diene ring makes dihedral angles of 1.12 (6) and 1.46 (6)° with the fused benzene rings. The dihedral angle between the two phenyl rings of the diphenyl­methyl­ene residue is 61.78 (6)°.

## Related literature

For the synthesis, see: Lewis & Glaser (2002 ▶). For the crystal structures of some aromatic azines, for example, fluorenone azine, see: Hagen *et al.* (1977 ▶). For the other heterocyclic aldehyde azines, see: Chen *et al.* (1995 ▶). For quadratic nonlinear optical properties, see: Wolff & Wortmann (1999 ▶).
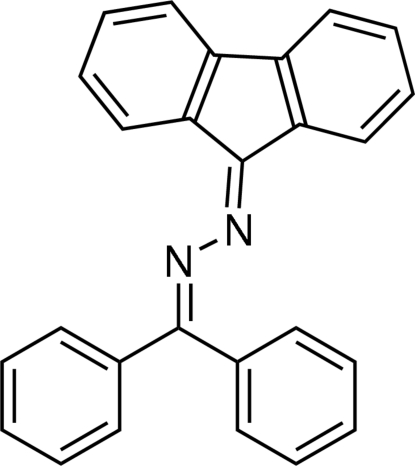


## Experimental

### 

#### Crystal data

C_26_H_18_N_2_
*M* *_r_* = 358.42
Monoclinic, 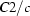

*a* = 22.8362 (3) Å
*b* = 13.1432 (2) Å
*c* = 12.4642 (2) Å
β = 92.874 (1)°
*V* = 3736.31 (10) Å^3^
*Z* = 8
Cu *K*α radiation
μ = 0.58 mm^−1^
*T* = 110 K
0.46 × 0.41 × 0.32 mm

#### Data collection

Oxford Xcalibur diffractometer with a Ruby Gemini detector
Absorption correction: multi-scan (*CrysAlis PRO*; Oxford Diffraction, 2009 ▶) *T*_min_ = 0.955, *T*_max_ = 1.000
7177 measured reflections
3682 independent reflections
3147 reflections with *I* > 2σ(*I*)
*R*_int_ = 0.019

#### Refinement

*R*[*F* ^2^ > 2σ(*F* ^2^)] = 0.037
*wR*(*F* ^2^) = 0.102
*S* = 1.06
3682 reflections
253 parameters
H-atom parameters constrained
Δρ_max_ = 0.23 e Å^−3^
Δρ_min_ = −0.20 e Å^−3^

### 

Data collection: *CrysAlis PRO* (Oxford Diffraction, 2009 ▶); cell refinement: *CrysAlis PRO*; data reduction: *CrysAlis PRO*; program(s) used to solve structure: *SHELXS97* (Sheldrick, 2008 ▶); program(s) used to refine structure: *SHELXL97* (Sheldrick, 2008 ▶); molecular graphics: *ORTEP-3* (Farrugia, 1997 ▶); software used to prepare material for publication: *PLATON* (Spek, 2009 ▶).

## Supplementary Material

Crystal structure: contains datablocks global, I. DOI: 10.1107/S160053681000070X/tk2612sup1.cif

Structure factors: contains datablocks I. DOI: 10.1107/S160053681000070X/tk2612Isup2.hkl

Additional supplementary materials:  crystallographic information; 3D view; checkCIF report

## Figures and Tables

**Fig. 1.**
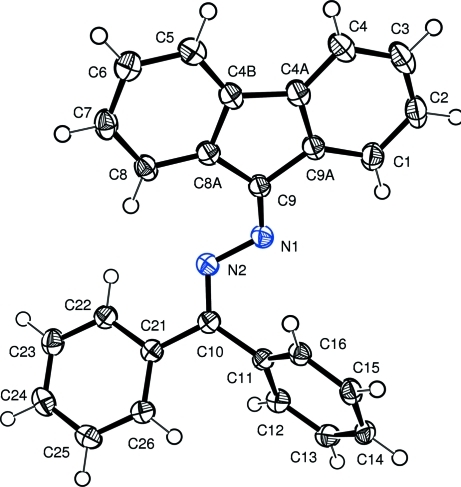
The molecular structure of the title compound, showing the atom-numbering scheme and displacement ellipsoids drawn at the 50% probability level. H atoms are shown as small spheres of arbitrary radius.

## supplementary crystallographic information

### Comment

Azines have received attention due to their unusual reactivity and spectral properties. For instance they are potential nonlinear optical (NLO) material. Molecular materials with quadratic nonlinear optical properties are currently attracting considerable interest (Wolff & Wortmann, 1999; Chen *et al.*, 1995). Some crystal structures are known (Hagen *et al.*, 1977). Optoelectronics has stimulated the search of highly nonlinear organic crystals for efficient signal processing. The title compound is an example of unsymmetrical fluorenone azine and shows a nonlinear optical behaviour. Herein, we report its crystal structure.

In the title molecule, C_26_H_18_N_2_, the 9*H*-fluorene unit is planar. The cyclopentadiene ring makes dihedral angles of 1.12 (6)° and 1.46 (6)° with the fused benzene rings. The dihedral angle between the two phenyl rings of the diphenylmethylene residue is 61.78 (6)°.

### Experimental

The compound was prepared in accord with literature precedents Lewis & Glaser (2002). The mixture of fluorenone hydrazone (1.94 g, 0.01 mol) and benzophenone (1.82 g, 0.01 mol) in ethanol with acetic acid was refluxed for 2 h. A mixture was cooled to room temperature over several hours. The solid obtained was separated, dried and then recrystallized from absolute ethanol. The yield of isolated product was (3.07 g, 78%).

### Refinement

H atoms were positioned geometrically and allowed to ride on their parent atoms, with C—H = 0.95 Å. *U*_iso_(H) = 1.2*U*_eq_(C).

### Crystal data

**Table:**

C_26_H_18_N_2_	*F*(000) = 1504
*M**_r_* = 358.42	*D*_x_ = 1.274 Mg m^−^^3^
Monoclinic, *C*2/*c*	Melting point: 377 K
Hall symbol: -C 2yc	Cu *K*α radiation, λ = 1.54184 Å
*a* = 22.8362 (3) Å	Cell parameters from 4987 reflections
*b* = 13.1432 (2) Å	θ = 5.1–73.9°
*c* = 12.4642 (2) Å	µ = 0.58 mm^−^^1^
β = 92.874 (1)°	*T* = 110 K
*V* = 3736.31 (10) Å^3^	Chunk, pale-yellow
*Z* = 8	0.46 × 0.41 × 0.32 mm

### Data collection

**Table:**

Oxford Xcalibur diffractometer with a Ruby Gemini detector	3682 independent reflections
Radiation source: Enhance (Cu) X-ray Source	3147 reflections with *I* > 2σ(*I*)
graphite	*R*_int_ = 0.019
Detector resolution: 10.5081 pixels mm^-1^	θ_max_ = 74.1°, θ_min_ = 5.2°
ω scans	*h* = −27→28
Absorption correction: multi-scan (*CrysAlis PRO*; Oxford Diffraction, 2009)	*k* = −10→15
*T*_min_ = 0.955, *T*_max_ = 1.000	*l* = −14→15
7177 measured reflections	

### Refinement

**Table:**

Refinement on *F*^2^	Primary atom site location: structure-invariant direct methods
Least-squares matrix: full	Secondary atom site location: difference Fourier map
*R*[*F*^2^ > 2σ(*F*^2^)] = 0.037	Hydrogen site location: inferred from neighbouring sites
*wR*(*F*^2^) = 0.102	H-atom parameters constrained
*S* = 1.06	*w* = 1/[σ^2^(*F*_o_^2^) + (0.0563*P*)^2^ + 1.6279*P*] where *P* = (*F*_o_^2^ + 2*F*_c_^2^)/3
3682 reflections	(Δ/σ)_max_ = 0.001
253 parameters	Δρ_max_ = 0.23 e Å^−^^3^
0 restraints	Δρ_min_ = −0.20 e Å^−^^3^

### Special details

**Table:**

Geometry. Bond distances, angles *etc*. have been calculated using the rounded fractional coordinates. All su's are estimated from the variances of the (full) variance-covariance matrix. The cell e.s.d.'s are taken into account in the estimation of distances, angles and torsion angles
Refinement. Refinement of *F*^2^ against ALL reflections. The weighted *R*-factor *wR* and goodness of fit *S* are based on *F*^2^, conventional *R*-factors *R* are based on *F*, with *F* set to zero for negative *F*^2^. The threshold expression of *F*^2^ > 2σ(*F*^2^) is used only for calculating *R*-factors(gt) *etc*. and is not relevant to the choice of reflections for refinement. *R*-factors based on *F*^2^ are statistically about twice as large as those based on *F*, and *R*- factors based on ALL data will be even larger.

### Fractional atomic coordinates and isotropic or equivalent isotropic displacement parameters (Å^2^)

**Table:**

	*x*	*y*	*z*	*U*_iso_*/*U*_eq_	
N1	0.35481 (4)	0.16268 (8)	0.34487 (8)	0.0276 (3)	
N2	0.30117 (4)	0.20980 (8)	0.32203 (8)	0.0266 (3)	
C1	0.48097 (6)	0.08981 (10)	0.38410 (10)	0.0325 (4)	
C2	0.54135 (6)	0.07751 (12)	0.40371 (11)	0.0381 (4)	
C3	0.57819 (6)	0.16072 (12)	0.41540 (11)	0.0385 (4)	
C4	0.55662 (6)	0.25969 (11)	0.40685 (10)	0.0329 (4)	
C4A	0.49686 (5)	0.27237 (10)	0.38678 (9)	0.0264 (3)	
C4B	0.46148 (5)	0.36564 (10)	0.37429 (9)	0.0252 (3)	
C5	0.47889 (5)	0.46657 (10)	0.37629 (9)	0.0291 (4)	
C6	0.43594 (6)	0.54174 (10)	0.36409 (10)	0.0309 (4)	
C7	0.37714 (6)	0.51540 (10)	0.35090 (9)	0.0295 (4)	
C8	0.35919 (5)	0.41379 (10)	0.34822 (9)	0.0262 (3)	
C8A	0.40189 (5)	0.33819 (9)	0.35879 (9)	0.0238 (3)	
C9	0.39839 (5)	0.22522 (9)	0.35779 (9)	0.0247 (3)	
C9A	0.45920 (5)	0.18833 (10)	0.37629 (9)	0.0266 (3)	
C10	0.25572 (5)	0.16399 (9)	0.35694 (9)	0.0225 (3)	
C11	0.25793 (5)	0.07200 (8)	0.42731 (9)	0.0215 (3)	
C12	0.22316 (5)	−0.01253 (9)	0.39994 (9)	0.0259 (3)	
C13	0.22468 (5)	−0.09829 (9)	0.46475 (10)	0.0281 (3)	
C14	0.25979 (5)	−0.09989 (9)	0.55906 (10)	0.0277 (3)	
C15	0.29376 (5)	−0.01569 (9)	0.58763 (9)	0.0264 (3)	
C16	0.29369 (5)	0.06937 (9)	0.52151 (9)	0.0233 (3)	
C21	0.19789 (5)	0.20994 (9)	0.32457 (9)	0.0226 (3)	
C22	0.19408 (5)	0.28377 (9)	0.24323 (9)	0.0257 (3)	
C23	0.14141 (6)	0.33068 (10)	0.21489 (10)	0.0319 (4)	
C24	0.09125 (6)	0.30600 (11)	0.26812 (11)	0.0348 (4)	
C25	0.09424 (5)	0.23372 (10)	0.34938 (10)	0.0307 (4)	
C26	0.14706 (5)	0.18576 (9)	0.37725 (9)	0.0251 (3)	
H1	0.45565	0.03265	0.37637	0.0390*	
H2	0.55735	0.01088	0.40909	0.0457*	
H3	0.61900	0.15017	0.42955	0.0462*	
H4	0.58209	0.31666	0.41454	0.0395*	
H5	0.51916	0.48422	0.38577	0.0350*	
H6	0.44700	0.61143	0.36480	0.0370*	
H7	0.34848	0.56765	0.34354	0.0355*	
H8	0.31883	0.39663	0.33940	0.0315*	
H12	0.19833	−0.01119	0.33649	0.0310*	
H13	0.20167	−0.15608	0.44465	0.0337*	
H14	0.26053	−0.15842	0.60383	0.0332*	
H15	0.31719	−0.01624	0.65280	0.0317*	
H16	0.31802	0.12588	0.54040	0.0279*	
H22	0.22823	0.30178	0.20708	0.0308*	
H23	0.13947	0.37987	0.15898	0.0382*	
H24	0.05507	0.33849	0.24892	0.0417*	
H25	0.06007	0.21698	0.38608	0.0369*	
H26	0.14866	0.13604	0.43264	0.0301*	

### Atomic displacement parameters (Å^2^)

**Table:**

	*U*^11^	*U*^22^	*U*^33^	*U*^12^	*U*^13^	*U*^23^
N1	0.0245 (5)	0.0310 (5)	0.0277 (5)	0.0046 (4)	0.0057 (4)	0.0066 (4)
N2	0.0237 (5)	0.0284 (5)	0.0279 (5)	0.0023 (4)	0.0034 (4)	0.0040 (4)
C1	0.0348 (7)	0.0340 (7)	0.0293 (6)	0.0097 (5)	0.0066 (5)	0.0068 (5)
C2	0.0384 (7)	0.0428 (8)	0.0334 (7)	0.0195 (6)	0.0056 (6)	0.0080 (6)
C3	0.0270 (6)	0.0556 (9)	0.0328 (7)	0.0157 (6)	0.0012 (5)	0.0020 (6)
C4	0.0252 (6)	0.0463 (8)	0.0273 (6)	0.0066 (5)	0.0011 (5)	−0.0002 (5)
C4A	0.0263 (6)	0.0356 (7)	0.0174 (5)	0.0066 (5)	0.0036 (4)	0.0022 (5)
C4B	0.0245 (5)	0.0346 (7)	0.0167 (5)	0.0055 (5)	0.0023 (4)	0.0011 (4)
C5	0.0276 (6)	0.0364 (7)	0.0233 (6)	0.0004 (5)	0.0003 (5)	−0.0011 (5)
C6	0.0379 (7)	0.0302 (6)	0.0245 (6)	0.0014 (5)	0.0011 (5)	−0.0001 (5)
C7	0.0343 (7)	0.0320 (7)	0.0224 (6)	0.0103 (5)	0.0023 (5)	0.0028 (5)
C8	0.0242 (5)	0.0336 (6)	0.0210 (5)	0.0069 (5)	0.0028 (4)	0.0044 (5)
C8A	0.0247 (6)	0.0314 (6)	0.0157 (5)	0.0036 (5)	0.0039 (4)	0.0034 (4)
C9	0.0240 (6)	0.0310 (6)	0.0195 (5)	0.0056 (4)	0.0060 (4)	0.0059 (4)
C9A	0.0254 (6)	0.0350 (7)	0.0198 (5)	0.0067 (5)	0.0055 (4)	0.0050 (5)
C10	0.0261 (6)	0.0219 (6)	0.0198 (5)	0.0001 (4)	0.0029 (4)	−0.0030 (4)
C11	0.0229 (5)	0.0207 (5)	0.0214 (5)	0.0021 (4)	0.0051 (4)	−0.0015 (4)
C12	0.0282 (6)	0.0255 (6)	0.0240 (5)	−0.0002 (5)	0.0022 (4)	−0.0043 (5)
C13	0.0291 (6)	0.0205 (6)	0.0354 (6)	−0.0017 (4)	0.0086 (5)	−0.0045 (5)
C14	0.0299 (6)	0.0221 (6)	0.0318 (6)	0.0065 (5)	0.0100 (5)	0.0046 (5)
C15	0.0270 (6)	0.0279 (6)	0.0245 (6)	0.0077 (5)	0.0026 (4)	0.0009 (5)
C16	0.0232 (5)	0.0220 (5)	0.0248 (6)	0.0016 (4)	0.0036 (4)	−0.0036 (4)
C21	0.0262 (6)	0.0213 (5)	0.0202 (5)	0.0000 (4)	0.0015 (4)	−0.0038 (4)
C22	0.0289 (6)	0.0251 (6)	0.0233 (6)	0.0005 (5)	0.0035 (4)	−0.0010 (4)
C23	0.0347 (7)	0.0315 (7)	0.0291 (6)	0.0053 (5)	−0.0012 (5)	0.0031 (5)
C24	0.0274 (6)	0.0375 (7)	0.0389 (7)	0.0072 (5)	−0.0029 (5)	−0.0018 (6)
C25	0.0241 (6)	0.0344 (7)	0.0339 (7)	−0.0012 (5)	0.0043 (5)	−0.0048 (5)
C26	0.0276 (6)	0.0245 (6)	0.0232 (5)	−0.0018 (4)	0.0029 (4)	−0.0029 (4)

### Geometric parameters (Å, °)

**Table:**

N1—N2	1.3893 (13)	C15—C16	1.3889 (16)
N1—C9	1.2946 (15)	C21—C22	1.4029 (16)
N2—C10	1.2940 (15)	C21—C26	1.3986 (16)
C1—C2	1.3976 (19)	C22—C23	1.3817 (18)
C1—C9A	1.3887 (18)	C23—C24	1.3906 (19)
C2—C3	1.383 (2)	C24—C25	1.3878 (19)
C3—C4	1.393 (2)	C25—C26	1.3896 (17)
C4—C4A	1.3851 (18)	C1—H1	0.9500
C4A—C4B	1.4721 (18)	C2—H2	0.9500
C4A—C9A	1.4020 (18)	C3—H3	0.9500
C4B—C5	1.3847 (18)	C4—H4	0.9500
C4B—C8A	1.4117 (16)	C5—H5	0.9500
C5—C6	1.3951 (18)	C6—H6	0.9500
C6—C7	1.3884 (19)	C7—H7	0.9500
C7—C8	1.3969 (19)	C8—H8	0.9500
C8—C8A	1.3938 (17)	C12—H12	0.9500
C8A—C9	1.4870 (17)	C13—H13	0.9500
C9—C9A	1.4780 (16)	C14—H14	0.9500
C10—C11	1.4931 (16)	C15—H15	0.9500
C10—C21	1.4893 (16)	C16—H16	0.9500
C11—C12	1.3979 (16)	C22—H22	0.9500
C11—C16	1.3966 (16)	C23—H23	0.9500
C12—C13	1.3862 (17)	C24—H24	0.9500
C13—C14	1.3895 (17)	C25—H25	0.9500
C14—C15	1.3877 (17)	C26—H26	0.9500
			
N1···C16	2.9339 (15)	C21···H7^vii^	2.9600
N2···C8	3.0012 (16)	C21···H16^v^	2.7700
N1···H1	2.8800	C22···H14^i^	2.8800
N1···H16	2.6600	C22···H16^v^	2.9700
N2···H8	2.5000	C23···H15^v^	3.0700
N2···H16	2.9400	C25···H16^v^	3.0100
N2···H22	2.4600	C26···H12	2.9000
N2···H14^i^	2.9100	C26···H16^v^	2.7800
C1···C1^ii^	3.4960 (18)	H1···N1	2.8800
C1···C2^iii^	3.4966 (19)	H1···C2^iii^	3.1000
C2···C1^iii^	3.4966 (19)	H2···C1^iii^	3.0600
C3···C9^ii^	3.5752 (18)	H3···C14^iii^	2.8400
C3···C15^iii^	3.4924 (18)	H3···C15^iii^	2.6800
C4···C9^ii^	3.5328 (17)	H3···H15^iii^	2.5300
C4A···C4A^ii^	3.4199 (16)	H4···C5	3.0900
C5···C5^ii^	3.3384 (16)	H5···C4	3.0800
C5···C5^iv^	3.3036 (16)	H5···C5^iv^	3.0300
C7···C14^v^	3.5528 (18)	H7···C21^vi^	2.9600
C7···C13^v^	3.5226 (17)	H7···C13^v^	3.0100
C8···N2	3.0012 (16)	H7···C14^v^	2.8400
C8A···C26^v^	3.5415 (16)	H8···N2	2.5000
C9···C4^ii^	3.5328 (17)	H8···H12^vi^	2.5200
C9···C3^ii^	3.5752 (18)	H12···C21	2.9100
C12···C26	3.1373 (17)	H12···C26	2.9000
C13···C7^v^	3.5226 (17)	H12···H26	2.5700
C14···C7^v^	3.5528 (18)	H12···C7^vii^	2.8500
C15···C3^iii^	3.4924 (18)	H12···C8^vii^	2.7700
C16···N1	2.9339 (15)	H12···H8^vii^	2.5200
C16···C22^v^	3.5100 (16)	H13···H22^vii^	2.6000
C16···C21^v^	3.4776 (16)	H14···N2^viii^	2.9100
C21···C16^v^	3.4776 (16)	H14···C22^viii^	2.8800
C22···C16^v^	3.5100 (16)	H14···H22^viii^	2.4200
C26···C8A^v^	3.5415 (16)	H15···H3^iii^	2.5300
C26···C12	3.1373 (17)	H15···C23^v^	3.0700
C1···H2^iii^	3.0600	H16···N1	2.6600
C2···H1^iii^	3.1000	H16···N2	2.9400
C4···H5	3.0800	H16···C21^v^	2.7700
C5···H4	3.0900	H16···C22^v^	2.9700
C5···H5^iv^	3.0300	H16···C25^v^	3.0100
C7···H12^vi^	2.8500	H16···C26^v^	2.7800
C8···H26^v^	2.8200	H22···N2	2.4600
C8···H12^vi^	2.7700	H22···H14^i^	2.4200
C8A···H26^v^	2.9200	H22···C12^vi^	3.0200
C11···H26	2.6400	H22···C13^vi^	2.7700
C12···H26	2.6300	H22···H13^vi^	2.6000
C12···H22^vii^	3.0200	H24···H24^ix^	2.5200
C13···H22^vii^	2.7700	H26···C11	2.6400
C13···H7^v^	3.0100	H26···C12	2.6300
C14···H7^v^	2.8400	H26···H12	2.5700
C14···H3^iii^	2.8400	H26···C8^v^	2.8200
C15···H3^iii^	2.6800	H26···C8A^v^	2.9200
C21···H12	2.9100		
			
N2—N1—C9	114.01 (10)	C22—C23—C24	120.09 (12)
N1—N2—C10	115.95 (10)	C23—C24—C25	119.76 (12)
C2—C1—C9A	117.82 (12)	C24—C25—C26	120.22 (11)
C1—C2—C3	121.10 (14)	C21—C26—C25	120.67 (11)
C2—C3—C4	121.29 (13)	C2—C1—H1	121.00
C3—C4—C4A	117.88 (13)	C9A—C1—H1	121.00
C4—C4A—C4B	130.53 (12)	C1—C2—H2	119.00
C4—C4A—C9A	121.08 (12)	C3—C2—H2	119.00
C4B—C4A—C9A	108.38 (10)	C2—C3—H3	119.00
C4A—C4B—C5	129.81 (11)	C4—C3—H3	119.00
C4A—C4B—C8A	108.77 (11)	C3—C4—H4	121.00
C5—C4B—C8A	121.41 (11)	C4A—C4—H4	121.00
C4B—C5—C6	118.48 (11)	C4B—C5—H5	121.00
C5—C6—C7	120.45 (12)	C6—C5—H5	121.00
C6—C7—C8	121.47 (12)	C5—C6—H6	120.00
C7—C8—C8A	118.44 (11)	C7—C6—H6	120.00
C4B—C8A—C8	119.72 (11)	C6—C7—H7	119.00
C4B—C8A—C9	107.90 (10)	C8—C7—H7	119.00
C8—C8A—C9	132.38 (11)	C7—C8—H8	121.00
N1—C9—C8A	132.51 (11)	C8A—C8—H8	121.00
N1—C9—C9A	121.43 (11)	C11—C12—H12	120.00
C8A—C9—C9A	106.06 (10)	C13—C12—H12	120.00
C1—C9A—C4A	120.82 (11)	C12—C13—H13	120.00
C1—C9A—C9	130.32 (12)	C14—C13—H13	120.00
C4A—C9A—C9	108.86 (11)	C13—C14—H14	120.00
N2—C10—C11	124.77 (10)	C15—C14—H14	120.00
N2—C10—C21	115.82 (10)	C14—C15—H15	120.00
C11—C10—C21	119.40 (10)	C16—C15—H15	120.00
C10—C11—C12	119.88 (10)	C11—C16—H16	120.00
C10—C11—C16	121.00 (10)	C15—C16—H16	120.00
C12—C11—C16	119.11 (10)	C21—C22—H22	120.00
C11—C12—C13	120.45 (10)	C23—C22—H22	119.00
C12—C13—C14	120.12 (11)	C22—C23—H23	120.00
C13—C14—C15	119.77 (11)	C24—C23—H23	120.00
C14—C15—C16	120.38 (11)	C23—C24—H24	120.00
C11—C16—C15	120.13 (11)	C25—C24—H24	120.00
C10—C21—C22	119.89 (10)	C24—C25—H25	120.00
C10—C21—C26	121.75 (10)	C26—C25—H25	120.00
C22—C21—C26	118.27 (10)	C21—C26—H26	120.00
C21—C22—C23	121.00 (11)	C25—C26—H26	120.00
			
C9—N1—N2—C10	−148.47 (11)	C4B—C8A—C9—C9A	1.37 (12)
N2—N1—C9—C8A	3.86 (18)	C8—C8A—C9—N1	1.5 (2)
N2—N1—C9—C9A	−176.38 (10)	C8—C8A—C9—C9A	−178.31 (12)
N1—N2—C10—C11	6.19 (17)	N1—C9—C9A—C1	−0.58 (19)
N1—N2—C10—C21	−175.08 (10)	N1—C9—C9A—C4A	179.70 (11)
C9A—C1—C2—C3	0.26 (19)	C8A—C9—C9A—C1	179.24 (12)
C2—C1—C9A—C4A	0.60 (18)	C8A—C9—C9A—C4A	−0.49 (12)
C2—C1—C9A—C9	−179.09 (12)	N2—C10—C11—C12	−130.13 (13)
C1—C2—C3—C4	−0.8 (2)	N2—C10—C11—C16	50.85 (17)
C2—C3—C4—C4A	0.35 (19)	C21—C10—C11—C12	51.19 (15)
C3—C4—C4A—C4B	179.63 (12)	C21—C10—C11—C16	−127.84 (12)
C3—C4—C4A—C9A	0.52 (18)	N2—C10—C21—C22	13.12 (16)
C4—C4A—C4B—C5	2.1 (2)	N2—C10—C21—C26	−163.26 (11)
C4—C4A—C4B—C8A	−177.76 (12)	C11—C10—C21—C22	−168.08 (10)
C9A—C4A—C4B—C5	−178.73 (12)	C11—C10—C21—C26	15.54 (17)
C9A—C4A—C4B—C8A	1.44 (13)	C10—C11—C12—C13	−179.81 (11)
C4—C4A—C9A—C1	−1.01 (18)	C16—C11—C12—C13	−0.77 (17)
C4—C4A—C9A—C9	178.74 (11)	C10—C11—C16—C15	177.99 (11)
C4B—C4A—C9A—C1	179.70 (11)	C12—C11—C16—C15	−1.05 (17)
C4B—C4A—C9A—C9	−0.55 (13)	C11—C12—C13—C14	1.60 (18)
C4A—C4B—C5—C6	−178.91 (11)	C12—C13—C14—C15	−0.61 (18)
C8A—C4B—C5—C6	0.90 (17)	C13—C14—C15—C16	−1.21 (17)
C4A—C4B—C8A—C8	178.01 (10)	C14—C15—C16—C11	2.04 (17)
C4A—C4B—C8A—C9	−1.72 (13)	C10—C21—C22—C23	−177.22 (11)
C5—C4B—C8A—C8	−1.84 (17)	C26—C21—C22—C23	−0.72 (17)
C5—C4B—C8A—C9	178.43 (10)	C10—C21—C26—C25	176.52 (11)
C4B—C5—C6—C7	0.37 (17)	C22—C21—C26—C25	0.08 (16)
C5—C6—C7—C8	−0.71 (18)	C21—C22—C23—C24	0.85 (19)
C6—C7—C8—C8A	−0.22 (17)	C22—C23—C24—C25	−0.3 (2)
C7—C8—C8A—C4B	1.46 (16)	C23—C24—C25—C26	−0.3 (2)
C7—C8—C8A—C9	−178.89 (11)	C24—C25—C26—C21	0.42 (19)
C4B—C8A—C9—N1	−178.85 (12)		

Symmetry codes: (i) *x*, −*y*, *z*−1/2; (ii) −*x*+1, *y*, −*z*+1/2; (iii) −*x*+1, −*y*, −*z*+1; (iv) −*x*+1, −*y*+1, −*z*+1; (v) −*x*+1/2, −*y*+1/2, −*z*+1; (vi) −*x*+1/2, *y*+1/2, −*z*+1/2; (vii) −*x*+1/2, *y*−1/2, −*z*+1/2; (viii) *x*, −*y*, *z*+1/2; (ix) −*x*, *y*, −*z*+1/2.
